# Analytical study on systemic lupus erythematosus and its association with cutaneous manifestations

**DOI:** 10.6026/973206300211627

**Published:** 2025-06-30

**Authors:** Nutheti Pavani, Himanshu Dua, Mohammed Zakiullah Shareef, Megha Joy, Trisha Hammigi, Sobiya Farook, Kosmita Karki

**Affiliations:** 1 Department of Dermatology, Madras Medical College, Chennai, Tamil Nadu, India; 2 Department of Pediatrics, N.K.P. Salve Institute of Medical Sciences & Research Centre and Lata Mangeshkar Hospital Nagpur, Maharashtra, India; 3 Department of Internal Medicine, Satyadev Multispeciality Hospital, Anakapalle Andhra Pradesh 531001, India; 4 Department of General Medicine, Queen Elizabeth The Queen Mother Hospital (QEQM), Margate, Kent CT9 4AN, UK; 5 Department of Obstetrics and Gynaecology, Madras Medical College, Chennai, Tamil Nadu, India; 6 Department of Medicine, East Lancashire Hospital NHS Trust, Royal Blackburn Hospital, Blackburn BB2 3HH, UK; 7 Department of Medicine, Nepal Manipal College of Medical Science, Fulbari, Pokhara - 11 , Kaski 33700, Nepal

**Keywords:** Systemic lupus erythematosus (SLE), cutaneous manifestations, disease activity, malar rash, SLE Disease Activity Index (SLEDAI)

## Abstract

Systemic lupus erythematosus (SLE) is a chronic multisystem autoimmune illness with cutaneous involvement being the most frequent of its clinical presentations. Therefore, it is of interest to determine the prevalence and patterns of cutaneous manifestations among SLE patients and their relationship with disease activity. Data comparison of 120 SLE patients was done based on demographic profile, nature of the cutaneous lesions and association with disease activity indices like the SLE disease activity index (SLEDAI). It was found that 75% of the patients presented with cutaneous involvement, of which malar rash was the most frequent manifestation, followed by discoid lesions and photosensitivity. Higher SLEDAI scores were significantly associated with more severe cutaneous symptoms (p < 0.01). The findings underscore the importance of early recognition and treatment of skin lesions for optimal disease outcome in SLE patients.

## Background:

Systemic lupus erythematosus is a chronic autoimmune disease characterized by immune dysregulation resulting in multisystem involvement and variable clinical presentations [1]. Cutaneous involvement is amongst the most common presentations of the disease and amongst the defining features of the disease is this cutaneous presentation [2]. Cutaneous manifestations have been reported in 60% to 85% of SLE patients and among the most common lesions, malar rash, discoid lesions and photosensitivity have been reported [3]. Apart from being a cause of morbidity in SLE, these manifestations are also important determinants of the severity and activity of the disease [4]. Pathogenesis of cutaneous SLE manifestation is a complex interaction of environmental, immunological and genetic factors [5]. Exposure to UV light, hormonal effects and deposition of immune complexes in the skin are known precipitating factors for an exacerbation of the lesions [6]. Not only do extensive cutaneous involvements in SLE result in discomfort and cosmetic deformity, they also may predict systemic exacerbations, with early diagnosis and treatment being absolutely essential. Although cutaneous lesions of SLE have clinical significance, the pattern and their correlation with activity have remained crudely poorly understood [7, 8]. Such a study would thus provide a systematic estimation of the incidence and types of cutaneous manifestation among SLE patients and assessment of their correlation with disease activity scores [9]. Trends and observations on the correlations may thus provide clues towards enhancing clinical management of SLE with special relevance to complications related to the skin.

## Materials and Methods:

An analytical observational study was undertaken for a year in a tertiary care hospital and included 120 patients diagnosed as having systemic lupus erythematosus by the 2019 EULAR/ACR classification criteria. Ethical permission was taken with informed consent taken from all individuals. Patients aged 18 years and more with confirmed SLE and manifestations of cutaneous involvement were recruited, while the exclusion criteria include patients with other autoimmune or dermatological conditions that would mimic SLE or those receiving immunosuppressive therapy less than six months. Data included demographic information, clinical histories and laboratory studies. The nature of skin lesions was classified into acute, subacute, or chronic, while disease activity used the SLE Disease Activity Index (SLEDAI). The statistical analysis was carried out using SPSS version 25 and the chi-square tests were used to determine the correlation between cutaneous manifestations and disease activity, considering p-value <0.05 as significant.

## Results:

Cutaneous manifestations were observed in 75% of the 120 SLE patients included in the study, with malar rash being the most common, followed by discoid lesions and photosensitivity. A significant association was noted between the severity of cutaneous manifestations and higher SLEDAI scores (p < 0.01). The demographic profile of the participants is showing a predominance of females (90%) and a mean age of 32.4 years, with most patients aged between 20 and 40 years. The distribution of cutaneous manifestations in the study population. Malar rash was the most frequent manifestation (60%), followed by photosensitivity (30%) and discoid lesions (25%). The correlation between cutaneous manifestations and disease activity, showing that patients with severe SLEDAI scores (>10) were more likely to have multiple or severe skin lesions compared to those with mild scores (≤10). The classification of skin lesions in SLE patients, with acute cutaneous lupus erythematosus (ACLE) being the most prevalent, observed in 50% of cases. The incidence of photo-sensitivity in patients with mild and severe disease activity.

Photosensitivity was significantly more common in patients with higher SLEDAI scores (p < 0.05). The prevalence of alopecia among SLE patients, highlight its association with higher disease activity scores. The impact of cutaneous manifestations on quality of life, with patients reporting a significant decline in dermatology life quality index (DLQI) scores as the severity of lesions increased. The association between specific cutaneous lesions and systemic involvement in SLE highlights a strong correlation between discoid lesions and renal manifestations (p < 0.05). The duration of disease in relation to the severity of cutaneous manifestations, with longer disease durations associated with chronic and severe skin lesions. The use of immunosuppressive therapy in patients with and without severe cutaneous involvement shows a higher reliance on combination therapies in those with severe lesions. The analytical study results on systemic lupus erythematosus (SLE) and its association with cutaneous manifestations are summarized in ten tables across, with critical findings. Table 1 (see PDF) provides the demographic profile of study participants, showing a predominance of females (90%) and a mean age of 32.4 years, with most patients aged between 20 and 40 years. Table 2 (see PDF) highlights the distribution of cutaneous manifestations, with malar rash (60%), photosensitivity (30%) and discoid lesions (25%) being the most prevalent. Table 3 (see PDF) demonstrates a significant correlation between severe cutaneous manifestations and higher SLEDAI scores (p < 0.01). Table 4 (see PDF) Classifies skin lesions as acute (50%), subacute (15%) and chronic (25%), as the presentation varies in SLE. Table 5 (see PDF) Looks at the incidence of photosensitivity, as it shows a higher incidence in patients with severe disease activity. Table 6 (see PDF) Points out the association of alopecia with the disease activity, showing a higher incidence in patients with a higher SLEDAI scores. Table 7 (see PDF) presents the impact of cutaneous manifestations on quality of life (QoL), with patients experiencing severe skin lesions reporting significantly lower QoL scores. Table 8 (see PDF) shows the correlation between specific cutaneous lesions and systemic involvement, with discoid lesions strongly associated with renal manifestations (p < 0.05). Table 9 (see PDF) explores the relationship between disease duration and the severity of skin lesions, indicating that chronic lesions are more common in patients with longer disease duration. Lastly, Table 10 (see PDF) illustrates the use of immunosuppressive therapy, with higher use of combination therapy noted in those with extensive cutaneous involvement. This summary indicates the clinical importance of cutaneous SLE disease, with prognostic and diagnostic significance and also the need for individualized treatment to address patients more effectively.

## Discussion:

This study presents the greatest responsibility of cutaneous manifestations in SLE and their close association with disease activity [10]. Cutaneous lesions occurred in 75% of patients, the most common of which were malar rash, discoid lesions and photosensitivity [11]. Findings are in agreement with earlier findings regarding the severity of cutaneous disease as an early and diagnostic feature of SLE [12]. SLEDAI scores correlated strongly with severe cutaneous lesions (p < 0.01) and we may presume skin lesions to be a marker of disease activity clinically [13]. The most common subtype found was acute cutaneous lupus erythematosus (ACLE) in 50% of the patients, with chronic lesions in patients having more prolonged disease duration [14]. The intense correlation of discoid lesions with renal disease implies the systemic character of SLE as well as the necessity of aggressive workup of patients presenting with specific skin lesions [15, 16]. It focuses on the skin-related symptoms of systemic lupus erythematosus (SLE) in a tertiary referral center [17]. The skin is one of the target organs most variably affected by the disease [18].

Acute cutaneous LE lesions included a butterfly rash with erythematous macules, telangiectasia or papulosquamous lesions [19]. This study also highlights the impact of cutaneous manifestations on quality of life, with higher dermatology life quality index (DLQI) scores in patients with severe skin lesions. The predominant use of combination immunosuppressive therapy in patients with severe cutaneous involvement underscores the difficulty of therapy in the treatment of such patients. In total, the results highlight the importance of early recognition and targeted treatment of skin lesions for improving outcomes in SLE patients. Future studies would focus on the study of new therapeutic approaches and preventive measures to minimize the impact of cutaneous disease on disease activity and patient quality of life.

## Conclusion:

The common and clinically relevant presence of skin manifestations in SLE, which are present in 75% of patients, is shown. Discoid rash, discoid lesions and photosensitivity were most common, evidencing active disease and diagnostic utility. Skin lesions are relevant to the quality of life; therefore early detection and direct treatment are warranted. Improved care strategies and emerging therapies need to be explored in order to optimize the outcome of SLE patients with cutaneous involvement.
